# Pathomechanisms of Disturbances Underlying Chronic Disorders

**DOI:** 10.3390/biomedicines12010131

**Published:** 2024-01-09

**Authors:** Dorota Formanowicz

**Affiliations:** Department of Medical Chemistry and Laboratory Medicine, Poznan University of Medical Sciences, Rokietnicka 8, 60-806 Poznań, Poland; doforman@ump.edu.pl

Chronic disorders’ complexity poses enormous challenges to our understanding of such disorders. It is essential to realize that the human body is a complex, sophisticated, and dynamic system whose homeostasis depends on the coherent cooperation of all systems at various levels to harmoniously respond to harmful stimuli. Chronic diseases arise due to the interactions between many overlapping factors. The best example is atherosclerosis, which begins with a sequence of immune–inflammatory reactions caused by multifactorial endothelial damage.

Atherosclerosis and other chronic diseases remain global health problems despite the enormous recent progress achieved in many areas of our lives. The latest technological and scientific achievements allow us to better understand the mechanisms underlying various diseases. Lifestyle changes, implementing new diagnostic standards based on increasingly sophisticated biomarkers, and introducing new personalized therapies targeting specific metabolic pathways have significantly influenced the effectiveness of treatment and the global epidemiological situation over the last few decades. However, despite this progress, chronic diseases and our understanding of them remain a challenge for medicine, and this Special Issue will address this problem. Its contains articles from various biological and medical science fields, discussing the pathomechanisms involved and recommending the treatment of the disturbances underlying chronic diseases from many perspectives.

One of the fundamental goals of systems biology and medicine is creating sufficiently accurately models of living organisms that enable us to precisely control them, which would be equivalent to applying a reliable treatment if dyshomeostasis affects the system (organism) [1,2]. There is growing belief in the possibility of making further significant progress in biological science and medicine using a novel approach that uses precise system analysis methods [3].

One of the crucial challenges facing systems biology and medicine is simulating the immune response [4], which is essential for understanding the basis and effective treatment of many diseases. Previous existing theoretical models either focused on specific cell–cell interactions or made certain parameter assumptions, which, combined with the wide variability in lymph node dimensions and numbers and patient-to-patient differences, made explicit quantitative modeling difficult [5,6,7]. To date, no theoretical model has been established that provides a consensus regarding the main cellular processes involved in the immune response. In the article by Scharf et al. (contribution 1), the researchers used the Petri net formalism to construct a semi-quantitative mathematical model of lymph nodes. The authors intended to develop a model that considers the perspectives of experienced pathologists and computer scientists in the field of systems biology. The developed model considers the immune response’s main cellular processes and meets the Petri net models’ formal requirements. This model provides a unique basis for further clinical, pharmacological, and machine learning research. The model presented by the authors can be extended and adapted to consider quantitative and qualitative human data inputs. These strategies may enable scientists to understand deeper processes in human lymph nodes and predict complications and outcomes of various therapies. Moreover, despite the system’s complexity, the modulation of cellular pathways in the model can explain certain aspects of the principles of cellular interactions, and the obtained results may help researchers to make therapeutic decisions.

The second study presented in the Special Issue is another fascinating project. In this study, Lubawy et al. (contribution 2) examined non-obvious connections between processes, consistent with the previously mentioned concept of systems and the holistic approach to the human body. The authors showed that the research results in patients with coexisting hyperuricemia were pro-oxidant and antioxidant factors [8,9] and a metabolic syndrome (MetS). Their results indicate potential relationships between two hormones (ghrelin and leptin) and inflammation in this group of patients. As the researchers emphasize, this study is the first study to approach the problem of urolithiasis in this way, the results of which may be helpful for enabling further research into appetite hormones and set new research trends.

It should be noted that there is increasing evidence that among the risk factors contributing to the development of atherosclerosis, the mentioned MetS [10,11] is a particularly strong and common predictor of cardiovascular events and their complications. The chronic, even low-grade inflammatory process associated with MetS has many harmful effects, promoting, among others, the activation of atherosclerotic plaque, which is responsible for clinical events.

The situation becomes more challenging when we deal with patients who have diffused atherosclerosis, although they do not always present well-known risk factors. One interesting project in this area is the study presented by Ilina et al. (contribution 3), in which the authors, via a single-center study, considered patients who underwent coronary artery bypass grafting (CABG) due to atherosclerosis. Based on the analysis of preoperative coronary angiography and intraoperative data results, the researchers classified the lesions as diffuse or focal, assigning the patient to one of the studied groups. Then, one year after CABG, they checked the number of major adverse cardiovascular events (MACE) in both groups. It should be noted that the groups did not differ in terms of lipid profile, with one exception: lipoprotein a (Lp(a)) concentration. The authors demonstrated that the risk of MACE was twice as high in patients with diffuse coronary artery disease and calcinosis than patients with segmental coronary artery disease. Moreover, they revealed an association between elevated Lp(a) and severe atherosclerotic phenotypes, including diffuse atherosclerosis and coronary artery calcification, independently of other well-known risk factors. It should be emphasized that the level of Lp(a) in human plasma is determined genetically, and currently, there is growing evidence of the involvement of Lp(a) in the processes of chronic inflammation in the vascular wall [12,13].

Another interesting topic discussed in this Special Issue is chronic pain, one of the most common diseases in the world. There are many types of chronic pain, and in their study, Navarro-Ledesma et al. (contribution 4) presented the results of research using the example of fibromyalgia (FM), a disease characterized by multi-site pain, fatigue, and sleep disorders. FM symptoms are very diverse and often suggest diseases of internal organs. Although FM is not life-threatening, it reduces its quality and impairs functioning [14]. Comprehensive treatment, including a healthy lifestyle, various rehabilitation methods, and pharmacotherapy, can significantly improve the patient’s comfort. In the aforementioned study, the authors analyzed daily changes in blood pressure (BP), the pain pressure threshold (PPT), and tissue elasticity in patients with fibromyalgia (FM) after whole-body photobiomodulation (PBM) treatment. This contribution is the first such study; therefore, comparing the obtained results to those of other studies was impossible. However, as the authors emphasized, further research is necessary to better understand the results obtained. This triple-blinded randomized clinical trial is part of the trend of considering new perspectives in the treatment of chronic diseases.

Subsequently, Baioccato et al. (contribution 5) focused on another issue related to chronic diseases, namely polycystic ovary syndrome (PCOS) [15,16], which affects an estimated 8–13% of women of reproductive age, up to 70% of whom are undiagnosed. PCOS is considered a female type of MetS since it is often associated with obesity, insulin resistance, abnormal lipid profile, and hypertension. In this study, the authors assessed the cardiorespiratory fitness (CRF) and strength levels of women with and without PCOS, which were matched for age, body composition, androgenic pattern, and insulinemia. Patients with and without PCOS were assessed for endocrine parameters, anthropometric parameters (using DEXA), and functional parameters (using cardiopulmonary exercise test and the handshake test), and physical parameters such as activity level (using the Global Physical Activity Questionnaire). However, there were no statistically significant differences between the studied parameters, besides cardiorespiratory fitness and strength. What is extremely interesting is that the group with PCOS had significantly better cardiorespiratory fitness and strength than the control group without PCOS. As the researchers concluded, the only determinant that could explain the observed differences was the presence of the PCOS itself. These results may suggest that PCOS diagnosis does not limit exercise capacity or exclude good functional performance, which is a fascinating observation.

Another chronic disease that continues to pose a challenge to the modern world is hepatitis C virus (HCV) infection [17]. As Popescu et al. (contribution 6) noted, HCV infection can be considered a current pandemic due to the widespread global occurrence of the HCV virus and its presence in a large undiagnosed population. The next study presented in this Special Issue is a continuation of research assessing patients with chronic HCV (40 patients with type 2 diabetes (T2DM) and 40 patients without T2DM) who received eight weeks of interferon-free treatment with direct-acting agents (DAA). Some had previously received interferon therapy, but not all reached a sustained virological response (SVR). After DAA treatment, all patients achieved SVR. Then, after three years, the non-invasive FibroMax test (composed of five different tests based on the selected biomarkers from serum) was performed. The study showed that the effective treatment of HCV infection might play an essential role in reducing fibrosis in these non-previously treated interferon patients with T2DM vs. those without T2DM. Moreover, FibroMax results showed a more significant reduction in T2DM patients than in those previously untreated patients. Diabetes mellitus is known to be associated with a high risk of atherosclerosis and cardiovascular disease; therefore, the extrahepatic effects of HCV eradication using DAA demonstrated in studies are promising.

As we begin to better understand the mechanisms underlying disease, we can expand the use of known drugs and pursue more individualized treatments. This is, for example, the case for sodium-glucose co-transporter 2 inhibitors (SGLT2i), which were initially used for the treatment of T2DM (a chronic and complex metabolic disease) and have since evolved from being a new hypoglycemic drug to being a potent drug with cardio- and reno-protective effects that represent a cornerstone of heart failure therapy. Moreover, despite initial safety concerns, they have recently emerged as an exciting independent therapeutic prospect for treating CKD, regardless of T2DM, as found by Skrabic et al. (contribution 7).

Nowadays, new treatment options for CKD, which is treated not only as a kidney disease but also as a chronic cardiovascular disorder [18], are being actively pursued. It is known that CKD is a complex multifactorial disease. Hypertension, altered lipid metabolism, diabetes, insulin resistance, and smoking, by enhancing oxidative stress and inflammation, not only affect both the cardiovascular and cerebrovascular systems but also contribute to CKD progression [19]. Still, regardless of the primary cause of renal tissue damage, tubulointerstitial fibrosis is a common pathomechanism, eventually leading to end-stage renal disease (ESRD). Many studies have shown that microRNAs (miRNAs, miRs), essential regulators of numerous processes, help to develop fibrosis and CKD. In the review in [20], the authors presented the roles of several miRNAs in the development of renal fibrosis and the potential pathways involved. This topic is crucial because reports of the effects of some miRNAs on fibrosis are contradictory, probably because miRNA expression and regulation occur in a tissue- and cell-dependent manner, and different populations were analyzed. Hence, extensive studies and clinical trials are needed to confirm the role of miRNAs in clinical settings. miRNAs have great potential; therefore, their analysis may improve diagnostic and therapeutic strategies. Various clinical trials currently use miRNAs in screening, diagnostics, and drug testing [21,22,23].

This Special Issue highlighted the need for further comprehensive research focusing on understanding the mechanisms underlying chronic diseases, as well as the usefulness of leveraging the achievements and opportunities offered by systems research to develop new therapeutic strategies for treating various aspects of chronic disorders.
